# Biohybrid -Se-S- Coupling Reactions of an Amino Acid Derived Seleninate

**DOI:** 10.3390/molecules18021963

**Published:** 2013-02-04

**Authors:** Mohannad Abdo, Zhexun Sun, Spencer Knapp

**Affiliations:** Department of Chemistry & Chemical Biology, Rutgers, the State University of New Jersey, 610 Taylor Road, Piscataway, NJ 08854, USA

**Keywords:** click reaction, biomimetic, seleninic acid, thiol

## Abstract

We describe the synthesis of the *N*-(2-seleninatoethyl) amide of *N*-Boc-phenylalanine, serving here as a peptide model, and its reductive coupling reactions under mild conditions with unprotected thiouridine and glutathione. Selenosulfide products such as these comprise reversibly conjugated bio-components, and can potentially find uses as probes of biological function, such as enzyme inhibitors, delivery systems, or structural mimics.

## 1. Introduction

Coupling reactions that join complex partners play a key role in biosynthesis as well as chemical synthesis. Chemists aspire to replicate in the laboratory the high efficiency and mild conditions achieved in Nature, and have developed a number of such reactions that succeed in aqueous solution at neutral pH and ambient temperature [1,2]. Unlike biosynthetic transformations, however, the chemical coupling reactions can embrace “unnatural” features such as uncommon bonds and unusual structures. The versatile azide-alkyne cycloaddition [3] exemplifies this feature: neither the azide nor the alkyne partner nor the triazole product would be considered biologically normal. Additionally, useful coupling reactions are often compatible with densely functionalized and generally unprotected partners incorporating, for example, peptide, nucleoside, carbohydrate, or oligomeric substructures [4,5]. The resulting biohybrid [6] products can feature useful biology-interactive properties such as (selected from among many recent examples) enzyme inhibitory activity [7,8,9], resistance to metabolic degradation [10], scaffolding [11,12], imaging and labeling [13], diagnostic features [14,15], special compartmentalization behavior [16], and drug delivery [17,18,19].

**Scheme 1:**
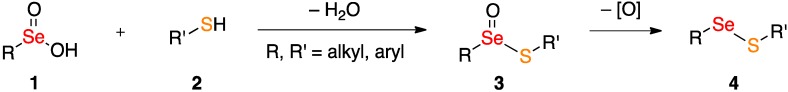
The redox coupling reaction of seleninates and thiols.

For several years our research group has investigated the redox coupling [20] of seleninic acids (RSeO_2_H, **1**, Scheme 1) with thiols **2** to give selenosulfides **4** [21,22,23]. A wide variety of biomimetic coupling partners can be assembled that incorporate the seleninic acid functionality as part of a carbohydrate, nucleoside, amino acid, or lipid framework [21]. The coupling reaction itself proceeds at room temperature in a wide variety of solvents, including water, alcohols, dioxane, THF, ethyl acetate, dichloromethane, acetonitrile, toluene, and DMF. The general mechanism of this reaction (Scheme 1) [20,24] proceeds initially through a thioseleninate intermediate **3**. Rapid reduction of **3** to the selenosulfide product **4** occurs in the presence of excess thiol (the byproduct is disulfide), or often with no obvious reducing agent, in which case the [O] from **3** “disappears” without reappearing as part of any isolable byproduct, even when close to 100% of the selenium and sulfur atoms are accounted for [21,23,24]. Despite this incomplete accounting, the conversion to selenosulfide **4** can be efficient for one-to-one mixtures of the two components. Air, moisture, buffers, and other additives do not seem to interfere [23]. The typical byproducts of the one-to-one coupling are the symmetrical disulfide and the symmetrical diselenide [23]. Exemplifying the potential of such couplings in bioorganic chemistry was the observation that a tyrosine phosphate mimic, in which a seleninate group (*i.e.*, Ar-CH_2_SeO_2_H) replaces phosphate (Ar-OPO_3_H_2_), effectively deactivated protein tyrosine phosphatase by forming a selenosulfide bond with the cysteine residue in the enzyme active site [22].

In order to expand on these studies, we chose to construct the seleninato moiety as a peptide model, and to examine its redox coupling to the sulfur atom of two relatively complex partners: the unprotected 4-thiouridine (a nucleoside thioamide), and glutathione (a tripeptide with an active thiol).

## 2. Results and Discussion

### 2.1. Synthesis of Seleninic Acid ***10***

**Scheme 2:**
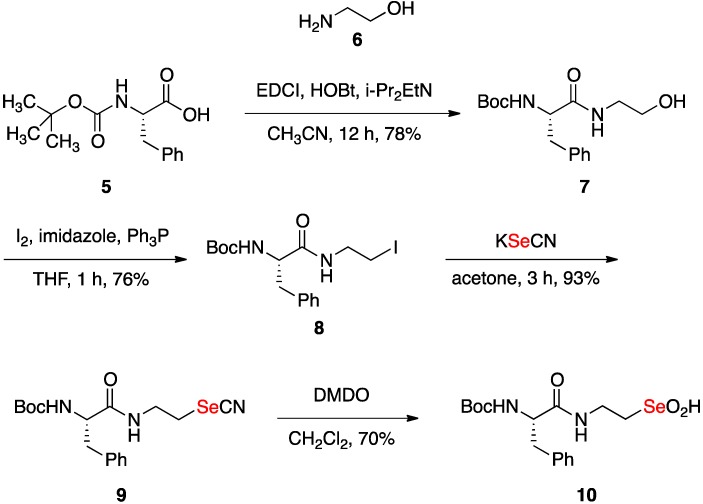
Synthesis of the seleninic acid **10** from *N*-(Boc)phenylalanine (**5**).

A greatly simplified “peptide” model compound was commercially available as *N*-(*tert*-butoxycarbonyl)-(*S*)-phenylalanine (**5**, Scheme 2). Standard amide formation with aminoethanol (**6**) mediated by the coupling reagent 1-[3-(dimethylamino)propyl]-3-ethylcarbodiimide hydrochloride (EDCI) gave the 2-hydroxylethylamide **7**. Conversion of **7** to the 2-iodoethylamide **8** occurred without incident, and then nucleophilic replacement of iodo by the selenocyanate anion led to **9**. Treatment of selenocyanate **9** with a slight excess of dimethyldioxirane (DMDO), our preferred reagent for the synthesis of seleninates [21], gave the stable seleninic acid **10**. Interestingly, in deuteriomethanol (CD_3_OD) solution **10** reversibly forms its apparent diasteriomeric seleninate trideuteriomethyl esters (1:1). Analogous behavior has been observed with nucleoside seleninic acids [25]. The structure of **10** is thus initially assigned based on the ^1^H-, ^13^C-, and ^77^Se- (1292.9 and 1294.1 ppm) NMR spectra of its trideuteriomethyl esters in deuteriomethanol solution, as well as the mass spectrum of the derived seleninate methyl ester with the appropriate Se isotope cluster centered on *m/z* 440 (the MNa^+^ ion of the methyl ester). The ^13^C-NMR spectra of **10** taken in acetone-*d_6_* or deuteriochloroform solution, however, showed the expected 12 signals for a single isomer of the seleninic acid (see Experimental).

**Scheme 3:**
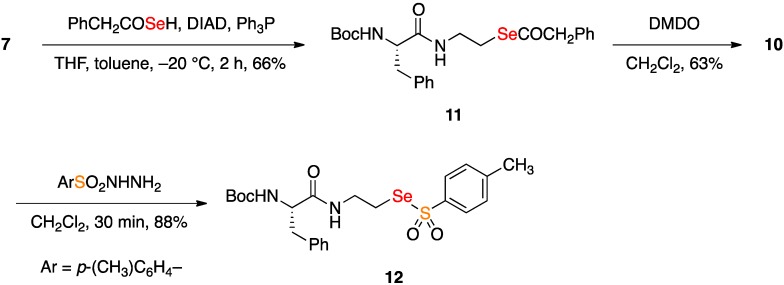
Alternative synthesis of seleninate **10** and confirmation of the structure.

The structure of **10** was additionally confirmed by an independent preparation (Scheme 3). Thus, 2-hydroxyethylamide **7** was converted to the selenoester **11** by use of the selenocarboxylate Mitsunobu reaction [26]. Oxidation with DMDO as before gave **10** in similar overall yield and purity. Further structural confirmation of **10** based on the rapid and efficient redox coupling reaction of seleninates with *p*-toluenesulfonylhydrazide [27,28] was carried out, leading to the selenosulfonate **12**. This diagnostic reaction of seleninates could qualify in some respects as a potentially useful biohybrid coupling reaction, although the biomimetic sulfonylhydrazide partners are typically not as complex nor are they as readily available as the thiols.

### 2.2. Coupling Reactions of Seleninic Acid ***10***

**Scheme 4:**
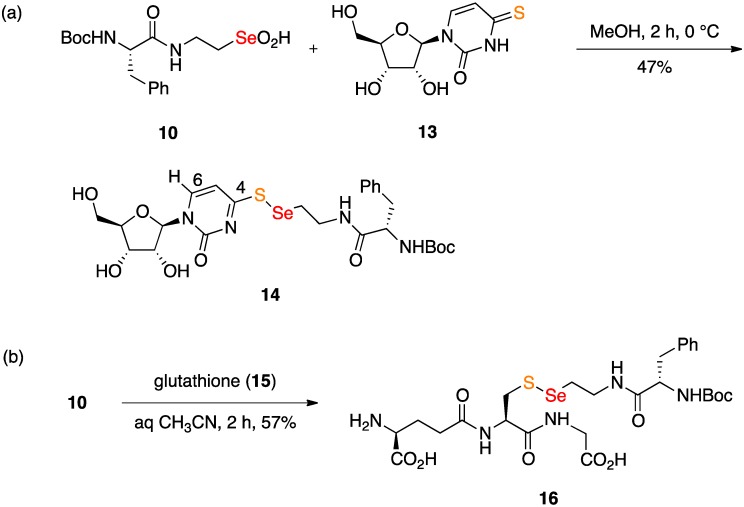
(**a**) Coupling reaction of seleninate **10** with 4-thiouridine. (**b**) Coupling reaction of **10** with glutathione.

The redox coupling reaction of seleninate **10** with commercial 4-thiouridine (**13**) was conveniently carried out in methanol solution, since both components easily dissolve (Scheme 4). Following our usual practice, **10** was added neat in one portion to a solution of **13** without any special precautions to keep the flask oxygen- or moisture-free. Chromatography of the product served to remove any disulfide and diselenide byproducts, and led to the modified nucleoside selenosulfide **14** in 47% yield. While selenosulfides can on occasion disproportionate to a mixture of the selenosulfide, diselenide, and disulfide, **14** was stable to storage. The 4-thioether structure for **14** is indicated by the pronounced downfield shift of H-6 to 8.52 ppm [29]. Sulfenylations of 4-thiouridine derivatives are known, and lead analogously to imidothioate *S*-derivatives (*i.e.*, disulfides) rather than *N*-substituted thioamides [29]. The coupled product **14** can be viewed as a reversibly modified nucleoside, since the Se-S bond can potentially be cleaved by reduction [30,31]; **14** can perhaps also serve as a simplified model for a peptide oligonucleotide hybrid.

The redox coupling reaction of seleninate **10** with commercial glutathione (GSH, **15**) was carried out in 1:1 acetonitrile/water, a solvent mixture that dissolved both reactants and the coupled product, selenosulfide **16** (Scheme 4). Reverse phase preparative HPLC served to purify **16**, which was characterized by its ^1^H and ^13^C-NMR spectra, as well as its protonated molecular ion possessing the required Se isotope cluster. Purified **16**, as for **14**, did not disproportionate on storage. Other types of selenylations of the GSH thiol have been reported recently, among these GSH pseudoglycosylation with a glycosyl diselenide [32], selenylation of GSH with ebselen [33], selenylation with selenocysteine [34], and selenylation of GSH with various electrophilic selenoallyl species [35].

## 3. Experimental

### 3.1. General

Organic solvents used for reactions were of reagent grade and were used as received. Flash chromatography was performed by using silica gel (E. Merck 230–400 mesh) as the stationary phase. Silica gel 60 F_254_ pre-coated plates were used for thin layer chromatography, and visualization was accomplished with UV light (254 nm) and iodine stain. Prep-scale reverse-phase chromatography was conducted with a Gilson 215 liquid handler/injector fitted with Gilson 333/334 binary HPLC pumps and UV/vis dual wavelength detector (model 156) and Trilution software. The chromatographies were carried out on a Waters XBridge Prep BEH 130 C18 5µm OBD 19 × 100mm column (part # 186003587). The eluent was acetonitrile (HPLC grade) and Millipore water with 0.05% formic acid buffer. The analytical LC-MS analyses were conducted by using a Waters 2767 sample manager, consisting of a Waters 2525 binary gradient HPLC connected to a diode array detector and a Waters Micromass ZQ mass spectrometer with electro-spray ionization. The LC-MS samples were analyzed as solutions in water or acetonitrile, prepared at 0.15–0.20 mg/mL concentration. The LC-MS chromatography was carried out on an Atlantis-C18 column (4.6 × 50 mm; 5 µm) with linear gradients of 0.05% formic acid in acetonitrile and 0.05% formic acid water. High-resolution mass spectrometry (HRMS) was obtained on Waters LC-TOF mass spectrometer (model LCT-XE Premier) by using electrospray ionization in positive or negative mode. ^1^H, ^13^C, and ^77^Se NMR spectra were obtained on a Varian UNITY 400 or 500 instrument. Chemical shifts (δ) are reported in parts per million (ppm) and are referenced to the residual solvent signal. Coupling constants (*J*) are reported in hertz (Hz). The usual abbreviations are used to describe multiplicities: s (singlet), d (doublet), t (triplet), q (quartet), br (broad). NMR solvents and all other commercially available reagents were used as received and without any further purification.

*(*S*)-tert-Butyl (1-((2-Hydroxyethyl)amino)-1-oxo-3-phenylpropan-2-yl)carbamate* (**7**) [36]. A solution of *N*-Boc-L-phenylalanine (**5,** 265.3 mg, 1.0 mmol) in of acetonitrile (8 mL) was stirred at 0 °C and then was treated sequentially with hydroxylbenzotriazole (184 mg, 1.2 mmol), diisopropylethylamine (388 mg, 3.0 mmol) and f 2-aminoethanol (6, 79 mg, 1.3 mmol). After 15 min at 0 °C, 1-ethyl-3-(3-dimethylaminopropyl)carbodiimide (230 mg, 1.2 mmol) was added and the reaction mixture was stirred for an additional 12 h at room temperature. The reaction was quenched with aqueous saturated sodium bicarbonate (10 mL). The reaction mixture was extracted with dichloromethane (3 × 10 mL). The combined organic extract was washed with brine, and then dried over sodium sulfate. The crude reaction product was purified by silica gel chromatography with 3% methanol in dichloromethane as the eluent to produce 240 mg (78%) of **7** as a colorless oil: ^1^H-NMR (500 MHz, CDCl_3_) δ 7.19–7.32 (m, 5 H), 6.62 (br s, 1 H), 5.30 (s, 1 H), 4.30–4.38 (m, 1 H), 3.50–3.63 (m, 2 H), 3.28–3.37 (m, 2 H), 3.00–3.09 (m, 2 H), 2.91 (br s, 1 H), 1.35 (s, 9 H); ^13^C-NMR (126 MHz, CDCl_3_) δ 172.7, 156.0, 137.1, 129.6, 128.7, 127.0, 80.3, 61.5, 56.3, 42.4, 39.2, 28.5; ESI-MS *m/z* 309.2 MH^+^.

*(*S*)-tert-Butyl (1-((2-Iodoethyl)amino)-1-oxo-3-phenylpropan-2-yl)carbamate* (**8**) [37]. A solution of **7** (240 mg, 0.785 mmol) in THF (6 mL) was combined with triphenylphosphine (411 mg, 1.57 mmol) and imidazole (106.8 mg, 1.57 mmol). After 5 min of stirring, iodine (398 mg, 1.57 mmol) was added and the reaction mixture was allowed to stir for an additional 1 h. The reaction mixture was concentrated and dissolved in dichloromethane (20 mL), which solution was washed sequentially with 5% aqueous Na_2_S_2_O_3_ (10 mL) and brine (10 mL), and then dried over sodium sulfate. Concentration and then chromatography with 3:7 ethyl acetate/hexanes as the eluent gave 248 mg (76%) of iodide **8** as a yellow solid: ^1^H-NMR (500 MHz, CDCl_3_) δ 7.20–7.36 (m, 5 H), 6.55 (br s, 1 H), 5.23 (br d, 1 H, *J* = 7.0 Hz), 4.29–4.38 (m, 1 H), 3.44–3.62 (m, 2 H), 3.02–3.18 (m, 4 H), 1.40 (s, 9 H); ^13^C-NMR (126 MHz, CDCl_3_) δ 171.6, 155.7, 136.9, 129.5, 128.9, 127.2, 80.4, 56.1, 42.0, 38.9, 28.6, 4.3; ESI-MS *m/z* 419.1 MH^+^.

*(*S*)-tert-Butyl (1-Oxo-3-phenyl-1-((2-selenocyanatoethyl)amino)propan-2-yl)carbamate* (**9**). A solution of iodide **8** (232 mg, 0.555 mmol) in acetone (3 mL) was treated with potassium selenocyanate (120 mg, 0.832 mmol). The reaction mixture was stirred for 3 h, concentrated, and then partitioned between dichloromethane and aqueous ammonia chloride. The organic extract was dried over sodium sulfate, concentrated, and then chromatographed with 3% methanol in dichloromethane as the eluent to afford 204 mg (93%) of **9** as a white solid: ^1^H-NMR (500 MHz, CDCl_3_) δ 7.18–7. 35 (m, 5 H), 6.55 (bs, 1 H), 5.10 (br d, 1 H, *J* = 7.0 Hz), 4.30–4.39 (m, 1 H), 3.65–3.74 (m, 1 H), 3.55-3.65 (m, 1 H), 3.10–3.17 (m, 1 H), 3.06 (app d, 2 H, *J* = 6.5 Hz), 2.94–3.03 (m, 1 H), 1.41 (s, 9 H); ^13^C-NMR (126 MHz, CDCl_3_) δ 172.3, 155.7, 136.8, 129.5, 129.0, 127.4, 101.6, 80.7, 56.1, 40.1, 38.6, 29.1, 28.5; ^77^Se NMR (95 MHz, CDCl_3_) δ 199.7 (*vs.* PhSeSePh at 460.0 ppm as an external standard); ESI-MS *m/z* 420.0 MNa^+^.

*(*S*)-2-(2-((tert-Butoxycarbonyl)amino)-3-phenylpropanamido)ethaneseleninic acid* (**10**). Dimethyldioxirane (DMDO) was added to a stirred solution of selenocyanate **9** (111 mg, 0.28 mmol) in dichloro-methane (7 mL) until starting material was consumed according to TLC analysis (total 1.05 mL of a 0.4 M solution of DMDO in moist acetone). The reaction mixture was concentrated and then chromatographed on silica gel with 8% methanol in dichloromethane as the eluent to give 77.3 mg (70%) of **10** as a colorless oil: ^1^H-NMR (500 MHz, CD_3_OD, as the two diasteriomeric seleninate—CD_3_ esters) δ 7.21–7.32 (m, 5 H), 4.22–4.31 (m, 1 H), 3.46–3.64 (m, 2 H), 3.04–3.12 (m, 2 H), 2.83–2.91 (m, 2 H), 1.38 (s, 9 H); ^13^C-NMR (126 MHz, CD_3_OD, as the two diasteriomeric seleninate—CD_3_ esters) δ 174.3 and 174.2, 156.4, 137.4, 129.3, 128.3, 126.7 and 126.6, 79.6, 57.2 and 57.0, 56.1 and 56.2, 38.0, 33.2 and 33.3, 27.5; ^13^C-NMR (126 MHz, acetone-*d_6_*) δ 173.3, 155.7, 138.2, 129.7, 128.5, 126.6, 78.8, 58.2, 56.2, 38.4, 33.7, 28.0; ^13^C-NMR (126 MHz, CDCl_3_) δ 173.9, 155.9, 136.9, 129.6, 128.9, 127.2, 80.5, 56.3, 55.9, 38.9, 33.6, 28.6; ^77^Se NMR (95 MHz, CD_3_OD, as the diasteriomeric seleninate esters, SeO_2_CD_3_) δ 1294.1, 1292.9 (*vs.* PhSeSePh at 460.0 ppm as an external standard); ESI-MS *m/z* 440.4 as the seleninate methyl ester···Na^+^.

*(*S*)-*Se*-(2-(2-((tert-Butoxycarbonyl)amino)-3-phenylpropanamido)ethyl) 2-phenylethaneselenoate* (**11**). A solution of triphenylphosphine (126 mg, 0.48 mmol) in tetrahydrofuran (1.5 mL) was stirred at −20 °C. Diisopropyl azodicarboxylate (97.1 mg, 0.48 mmol) was added dropwise and the reaction mixture was maintained at −20 °C until the white phosphonium intermediate formed. The reaction mixture was then cooled to −50 °C, and a solution of alcohol **7** (74.0 mg, 0.24 mmol) in tetrahydrofuran (2 mL) was added dropwise. After 5 min of stirring, a toluene solution (2 mL) of (2-phenyl)-selenoacetic acid [prepared by heating at reflux a 2 mL toluene solution of 100 mg (0.734 mmol) of phenyl acetic acid and 117 mg of Woollins’s reagent for 2 h] was added by cannula and the reaction mixture was allowed to warm from −50 to 23 °C, and then was stirred for an additional 2 h. The solution was concentrated and then chromatographed with 3:7 ethyl acetate/hexanes as the eluent to afford 77 mg (66%) of **11** as a colorless oil: ^1^H-NMR (500 MHz, CDCl_3_) δ 7.39–7.30 (m, 4 H), 7.30–7.25 (m, 4 H), 7.17 (d, 2 H, *J* = 7.0 Hz), 6.24 (bs, 1 H), 5.18 (bs, 1 H), 4.30 (bs, 1 H), 3.83 (s, 2 H), 3.33–3.47 (m, 2 H), 3.01 (br d, 2 H, J = 6.0 Hz), 2.77–2.92 (m, 2 H), 1.41 (s, 9 H); ^13^C-NMR (126 MHz, CD_3_OD) δ 200.7, 171.4, 155.8, 136.9, 132.9, 130.2, 129.6, 129.5, 129.0, 128.9, 128.8, 128.0, 127.1, 80.4, 56.2, 54.3, 40.1, 39.0, 28.5, 25.1; ^77^Se NMR (95 MHz, CDCl_3_) δ 547.6 (*vs.* PhSeSePh at 460.0 ppm as an external standard); ESI-MS *m/z* 513.1 MNa^+^.

*(*S*)-2-(2-((*tert*-Butoxycarbonyl)amino)-3-phenylpropanamido)ethaneseleninic acid* (**10**) *by oxidation of* **11**. Dimethyldioxirane was added to a stirred solution of selenoester **11** (43.4 mg, 0.089 mmol) in dichloromethane (1 mL) until starting material was consumed according to TLC analysis (total 0.488 mL of 0.4 M solution of DMDO in moist acetone). The reaction mixture was concentrated and then chromatographed on silica with 8% methanol in dichloromethane as the eluent to give 22.6 mg (63%) of **10** as a colorless oil that spectroscopically matched **10** as prepared above.

*(*S*)-*Se*-(2-(2-((*tert*-Butoxycarbonyl)amino)-3-phenylpropanamido)ethyl) 4-methylbenzenesulfonoselenoate* (**12**). A solution of seleninic acid **10** (22.5 mg, 0.056 mmol) in dichoromethane (3 mL) was added dropwise to a solution of *p*-toluenesulfonylhydrazide (11.3 mg, 0.061 mmol) in dichloromethane (1 mL). After 30 min, the reaction mixture was concentrated then and chromatographed on silica with 3:7 ethyl acetate/hexanes as the eluent to give 25.9 (88%) of selenosulfonate **12** as a yellow oil: ^1^H-NMR (500 MHz, CDCl_3_) δ 7.75 (d, 2 H, *J* = 8.0 Hz), 7.35 (d, 2 H, *J* = 8.0 Hz), 7.29 (t, 2 H, *J* = 7.0 Hz), 7.24 (t, 1 H, *J* = 7.0 Hz), 7.19 (d, 2 H, *J* = 7.0 Hz), 6.44 (br s, 1 H), 5.08 (br s, 1 H), 4.29–4.36 (m, 1 H), 3.55–3.66 (m, 2 H), 3.19–3.27 (m, 1 H), 3.11–3.19 (m, 1 H), 3.06 (br d, 2 H, *J* = 6.5 Hz), 2.45 (s, 3 H), 1.40 (s, 9 H); ^13^C-NMR (126 MHz, CDCl_3_) δ 171.9, 155.8, 145.2, 144.1, 136.9, 130.1, 129.5, 128.9, 127.3, 126.8, 82.3, 56.2, 39.4, 38.8, 33.0, 28.5, 21.9; ^77^Se NMR (95 MHz, CDCl_3_) δ 856.9 (*vs.* PhSeSePh at 460.0 ppm as an external standard); ESI-MS *m/z* 549.1 MNa^+^.

tert-Butyl (1-((2-(((1-((2R,3R,4S,5R)-3,4-Dihydroxy-5-(hydroxymethyl)tetrahydrofuran-2-yl)-2-oxo-1,2-dihydropyrimidin-4-yl)thio)selanyl)ethyl)amino)-1-oxo-3-phenylpropan-2-yl)carbamate (**14**). A solution of 4-thiouridine (**13**, 11.9 mg, 0.0456 mmol) in methanol (0.5 mL) was stirred at 0 °C and then was treated with seleninic acid **10** (18.4 mg, 0.0456 mmol) in one portion. After 2 h at 0 °C, the reaction was concentrated and then chromatographed with 5% methanol in dichloromethane as the eluent to produce 13.9 mg (47%) of selenosulfide **14** as a colorless oil: R_f_ 0.28 (5% methanol in dichloromethane); ^1^H-NMR (400 MHz, CD_3_OD) δ 8.52 (d, 1 H, J = 7.2 Hz), 7.27 (br s, 4 H), 7.21 (br s, 1 H), 6.89 (d, 1 H, J = 7.2 Hz), 5.87 (br s, 1 H), 4.27–4.32 (m, 1 H), 4.09–4.21 (m, 3 H), 3.81 and 3.97 (ABq, 2 H, J = 10.0 Hz), 3.70–3.78 (m, 1 H), 3.22–3.30 (m, 1 H), 3.07–3.15 (m, 2 H), 2.95–3.04 (m, 1 H), 2.86 (dd, 1 H, J = 13.2, 10.0 Hz), 1.31 (s, 9 H); ^13^C-NMR (126 MHz, CD_3_OD) δ 179.1, 174.7, 157.7, 156.1, 144.3, 138.8, 130.4, 129.5, 127.7, 103.6, 93.4, 85.9, 80.6, 76.6, 69.9, 61.1, 57.9, 39.4, 39.1, 33.5, 28.7; HRMS m/z 631.1367 MH^+^, calcd for C_25_H_35_N_4_O_8_SSe 631.1341; NI-HRMS m/z 629.1191 MH^−^, calcd for C_25_H_33_N_4_O_8_SSe 629.1184.

(6S,14R,19S)-19-Amino-6-benzyl-14-((carboxymethyl)carbamoyl)-2,2-dimethyl-4,7,16-trioxo-3-oxa-12-thia-11-selena-5,8,15-triazaicosan-20-oic acid (**16**). A stirred solution of glutathione (**15**, 15.2 mg, 0.0496 mmol) in acetonitrile/water (1:1, 2 mL) was treated with seleninic acid **10** (20.0 mg, 0.0496 mmol) in one portion. After 2 h, the resulting solution was directly purified by reverse-phase chromatography (gradient 5–60% acetonitrile over 10 min) and then lyophilized to give 19.0 mg (57%) of **16** as a white amorphous powder showing a single peak at 3.28 min by HPLC analysis. ^1^H-NMR (500 MHz, CD_3_OD) δ 7.17–7.29 (m, 5 H), 4.68 (dd, 1 H, J = 9.5, 4.5 Hz), 4.20–4.28 (m, 1 H), 4.02 (t, 1 H, J = 6.5 Hz), 3.93 (br s, 2 H), 3.52–3.62 (m, 1 H), 3.42–3.52 (m, 1 H), 2.96–3.10 (m, 3 H), 2.80–2.96 (m, 3 H), 2.58 (t, 2 H, J = 6.5 Hz), 2.12–2.29 (m, 2 H), 1.35 (s, 9 H); ^13^C-NMR (126 MHz, CD_3_OD) δ 173.3, 173.2, 171.7, 171.5, 170.4, 156.4, 137.4, 129.2, 128.3, 126.6, 79.5, 56.4, 53.7, 52.4, 40.7, 39.0, 38.3, 33.3, 31.2, 30.0, 27.5, 25.9; ESI-MS m/z 678.1 MH^+^.

## 4. Conclusions

Densely functionalized and unprotected coupling partners (4-thiuridine and glutathione) participate in the seleninic acid–thiol redox coupling reaction with the 2-seleninatoethyl amide **10** under mild and neutral conditions in protic solvents. The selenosulfide products **14** and **16**, while formed in modest yield, are stable and easily characterized. No obvious impediment prevents analogous coupling reactions between peptide-based seleninic acids and thiol-bearing peptides, proteins, or oligonucleotides, and such applications may now be contemplated.

## Supplementary Materials

NMR spectra for compounds **8**–**12**, **14**, and **16**, and HPLC trace for **16**.

Supplementary materials can be accessed at: http://www.mdpi.com/1420-3049/18/2/1963/s1.
