# Supplementary material for: Biohybrid -Se-S- Coupling Reactions of an Amino Acid Derived Seleninate
Source: Molecules. 2013 Feb 4;18(2):1963–72. doi: 10.3390/molecules18021963 (PMC6270073; doi:10.3390/molecules18021963)

Supplementary Materials

Figure S1. NMR Spectra for Compound **8**.

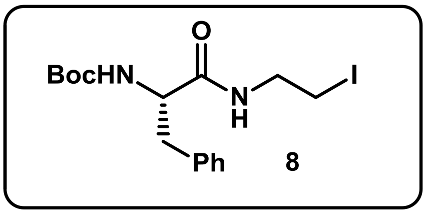

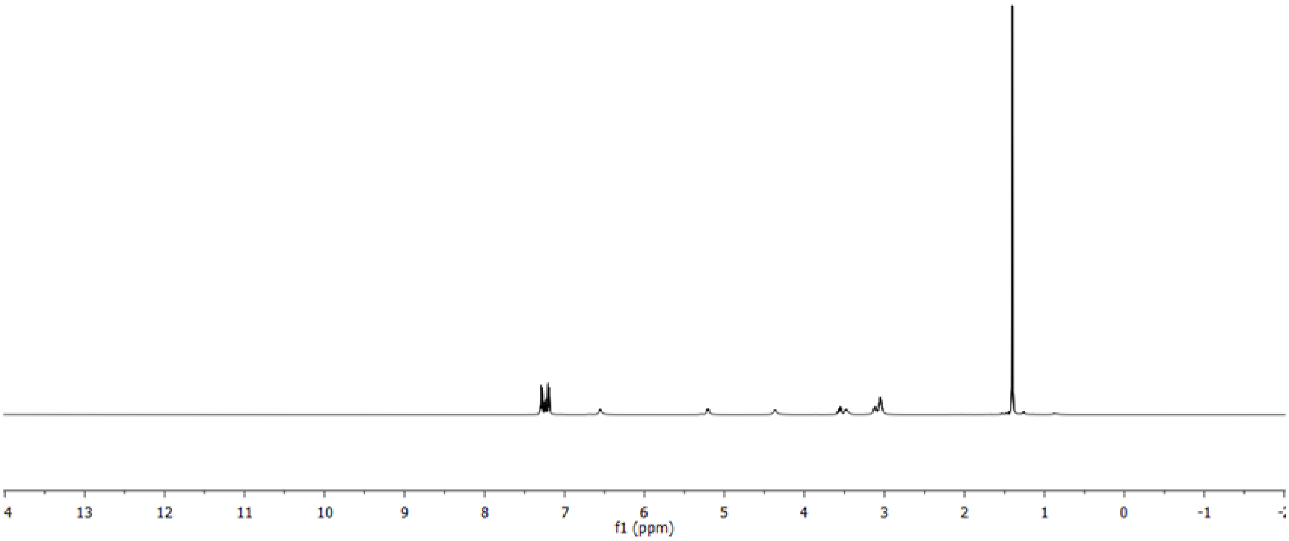

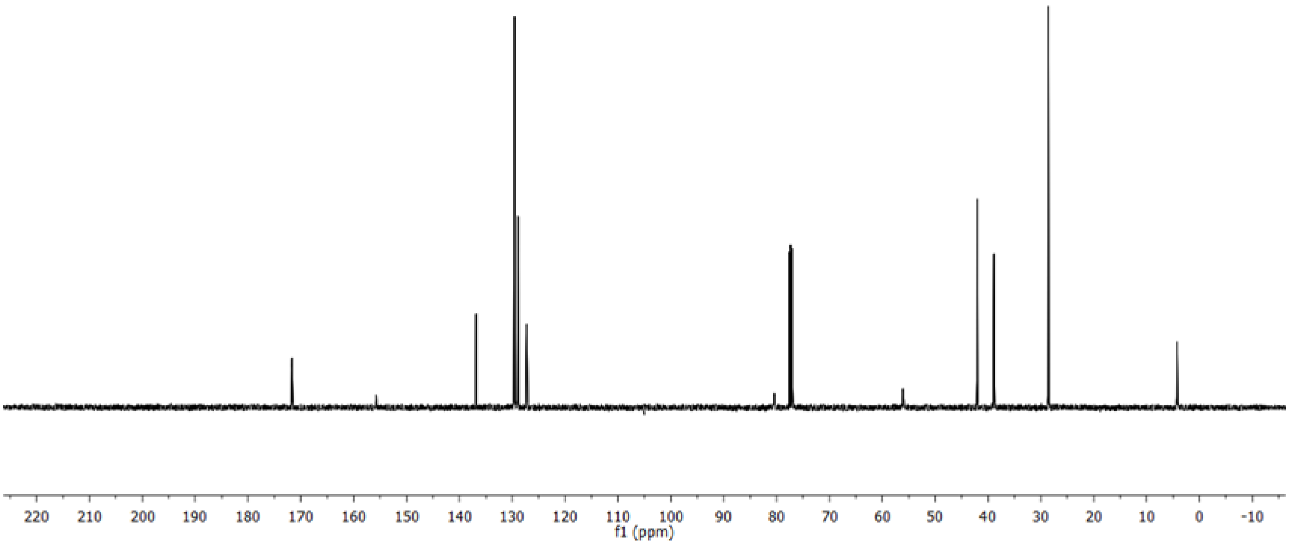

Figure S2. NMR Spectra for Compound **9**.

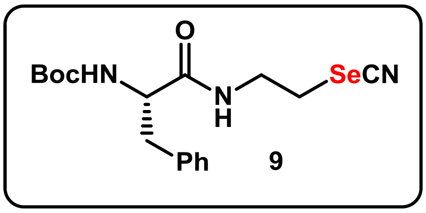

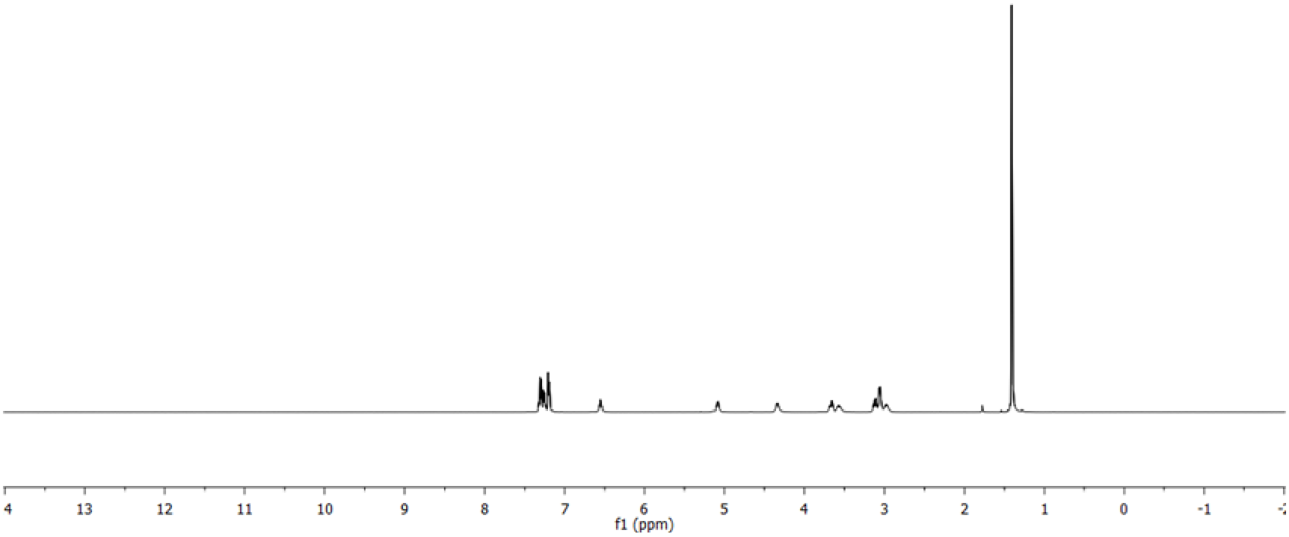

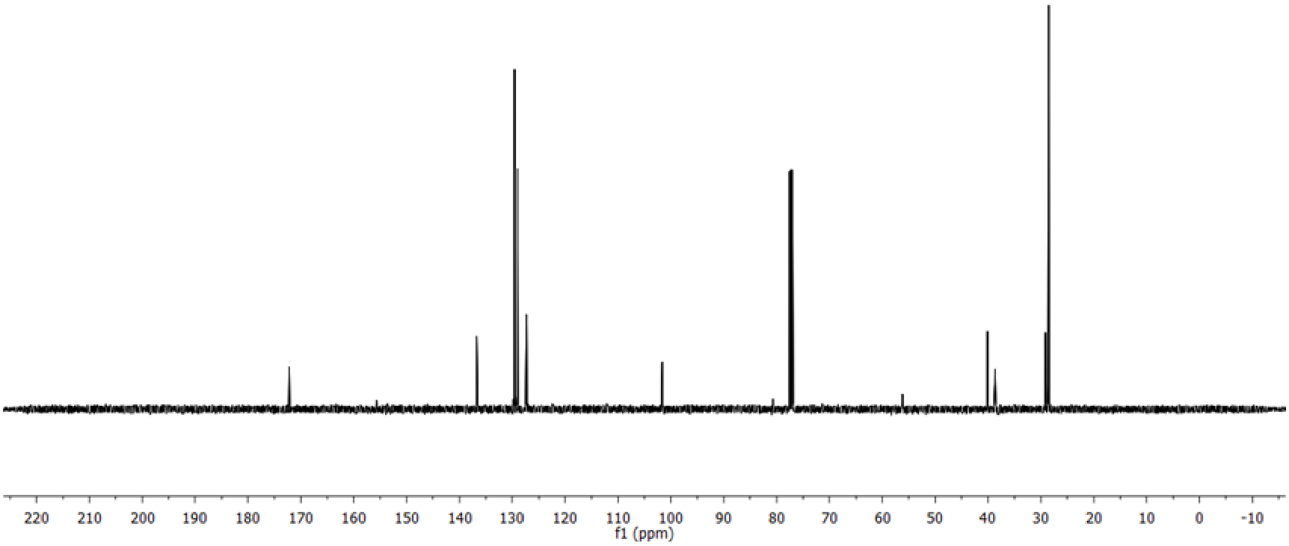

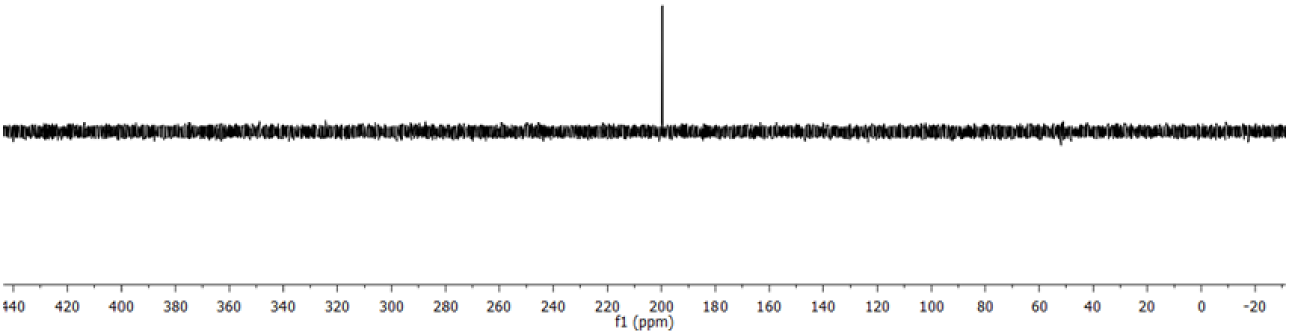

Figure S3. NMR Spectra for Compound **10**.

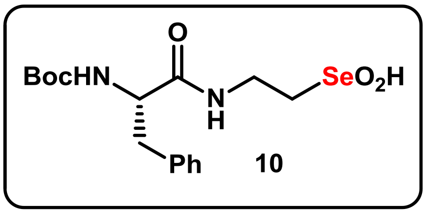

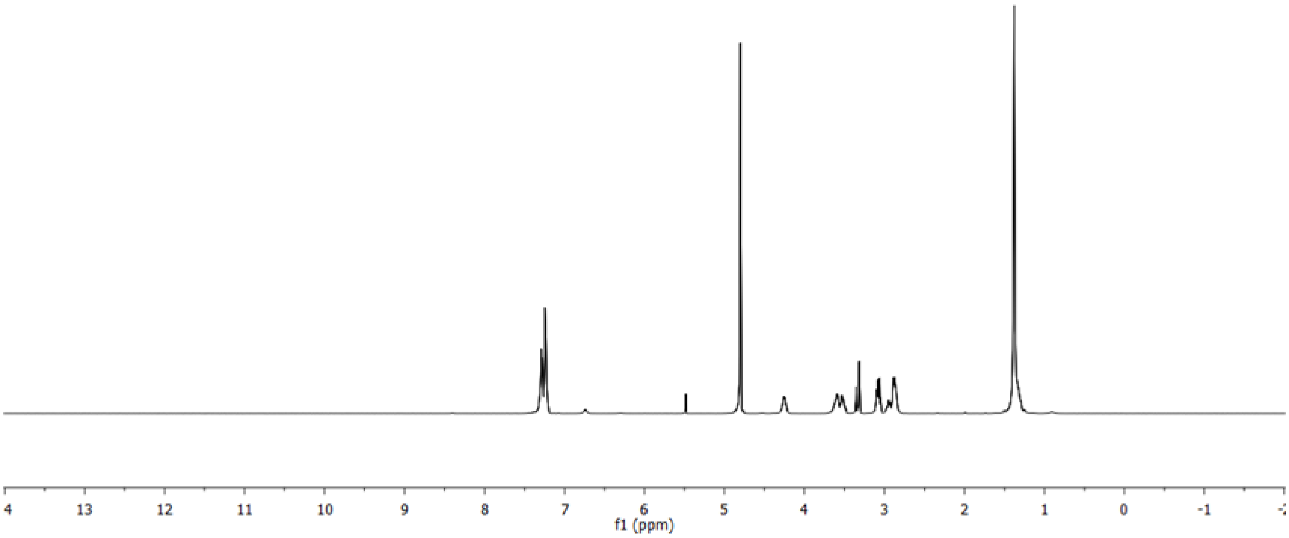

^{13}C-NMR of Compound **10** in CD_3OD.

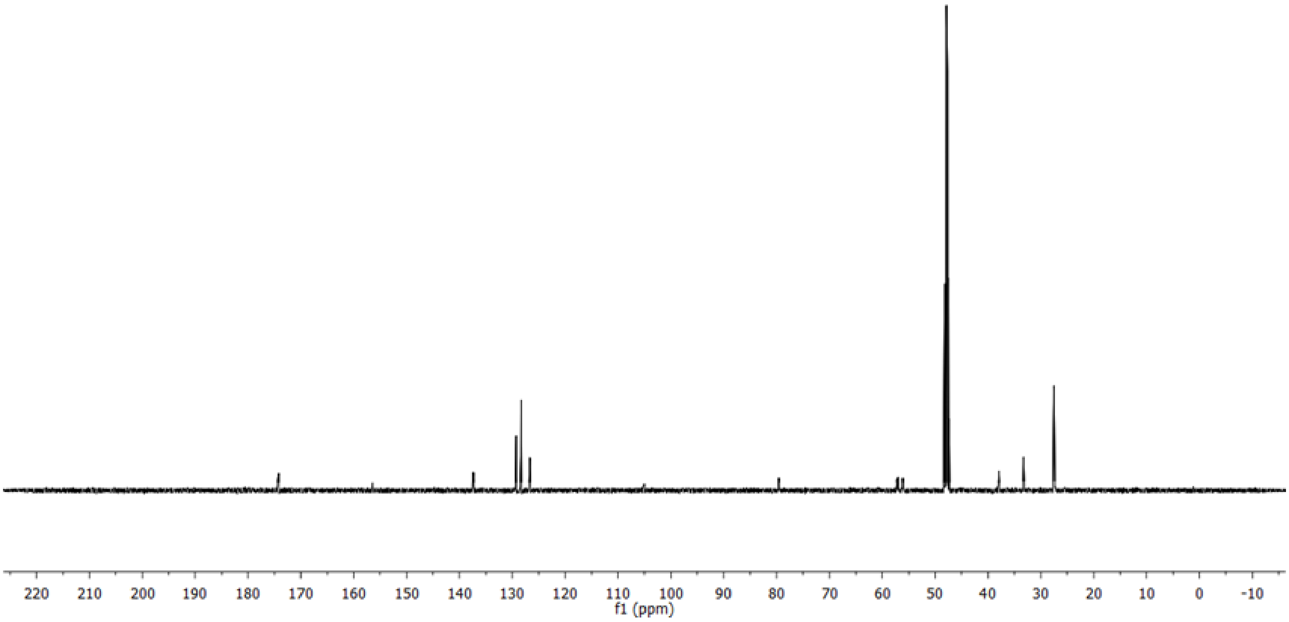

Figure S3. *Cont.*

^{13}C NMR of Compound **10** in Acetone-d_6.

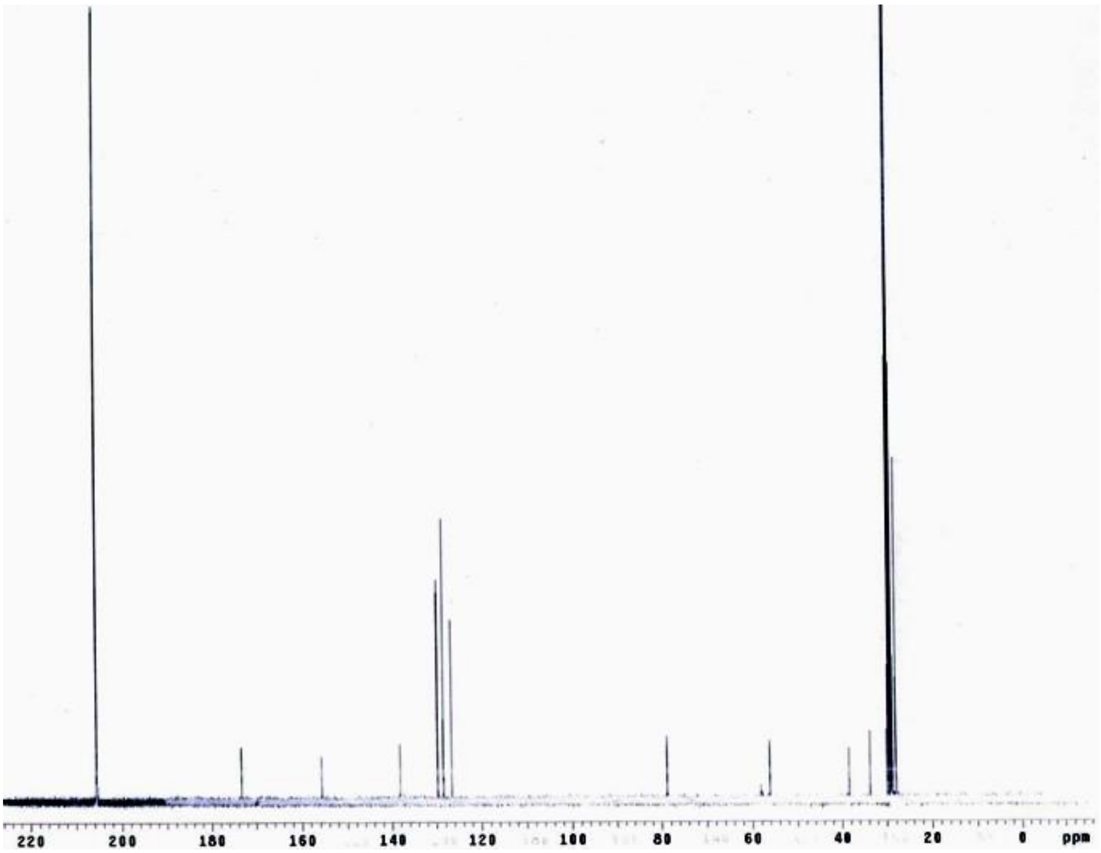

^{13}C-NMR of Compound **10** in $CDCl_3$.

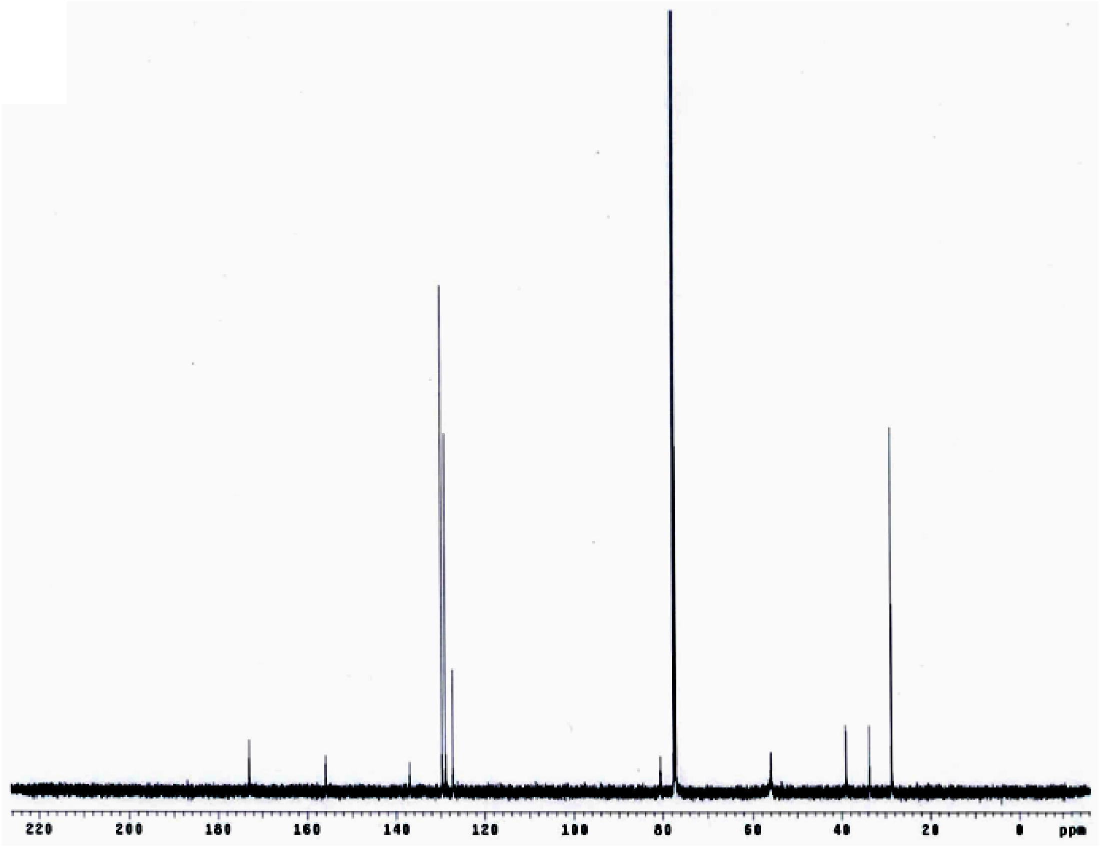

Figure S3. *Cont.*

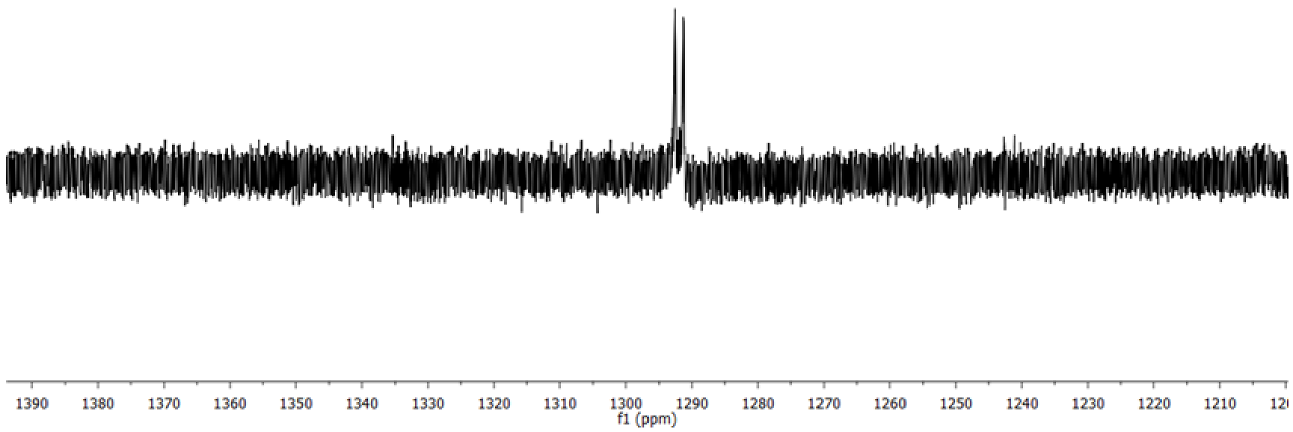

Figure S4. NMR Spectra for Compound **11**.

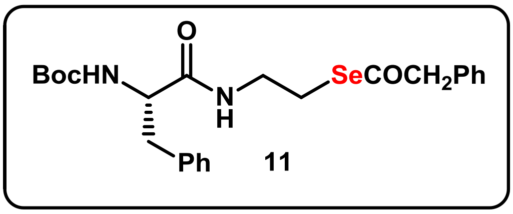

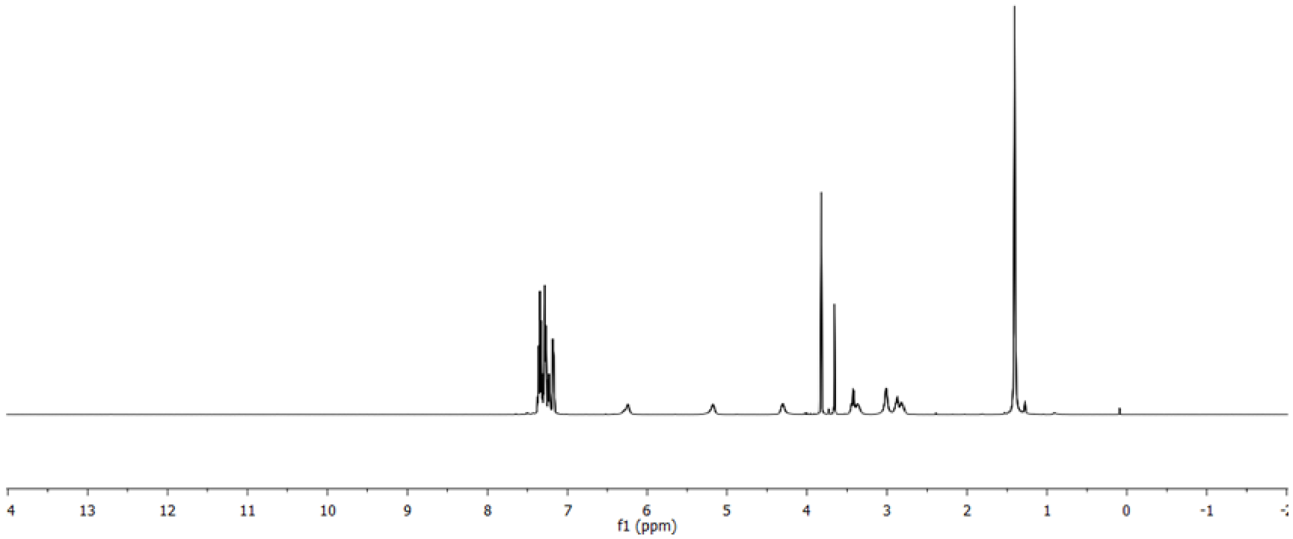

Figure S4. *Cont.*

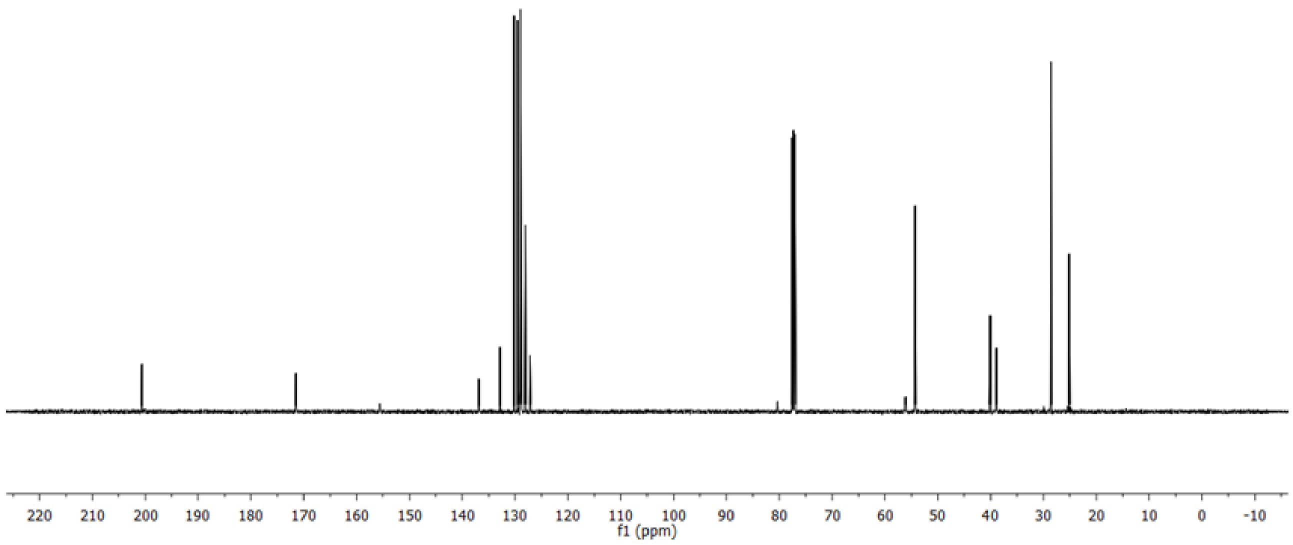

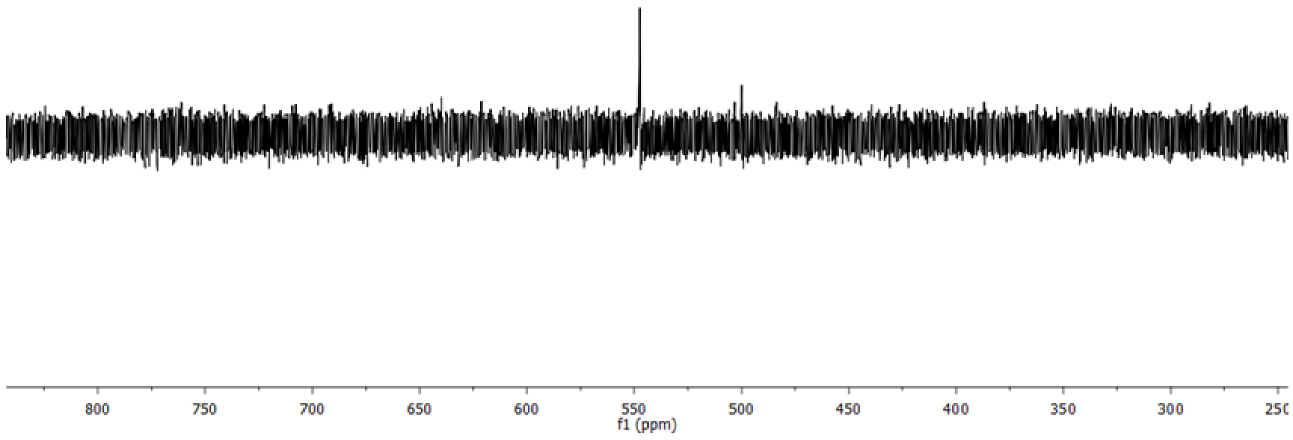

Figure S5. NMR Spectra for Compound **12**.

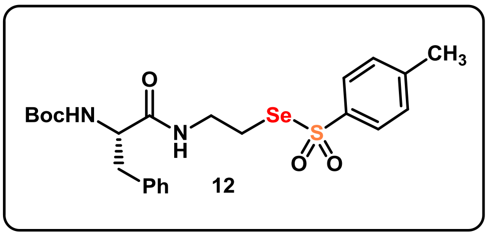

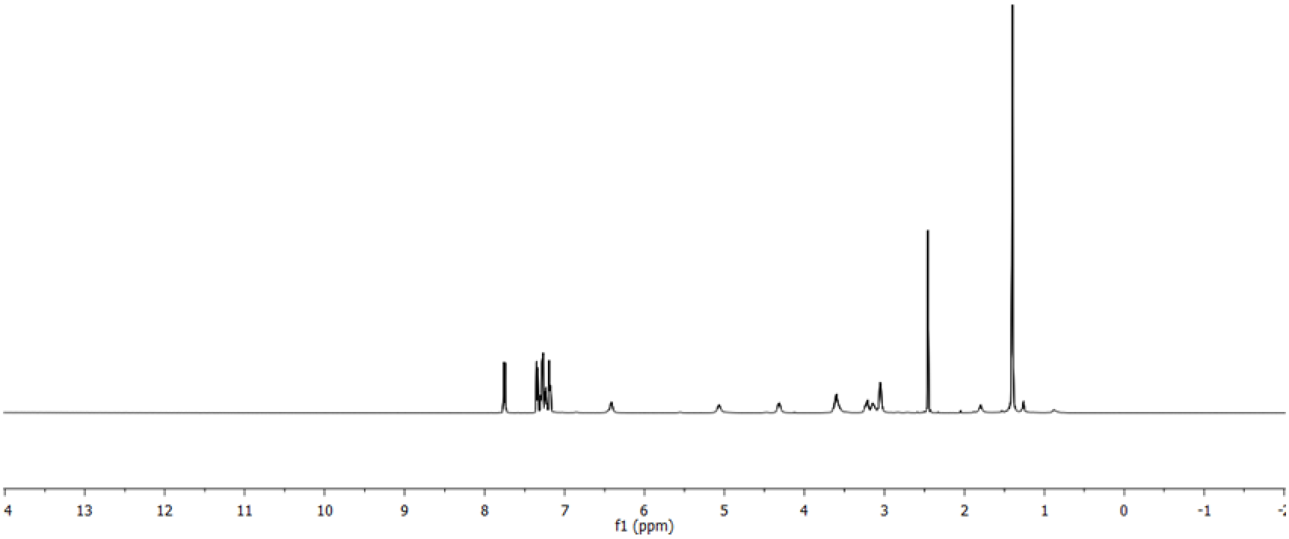

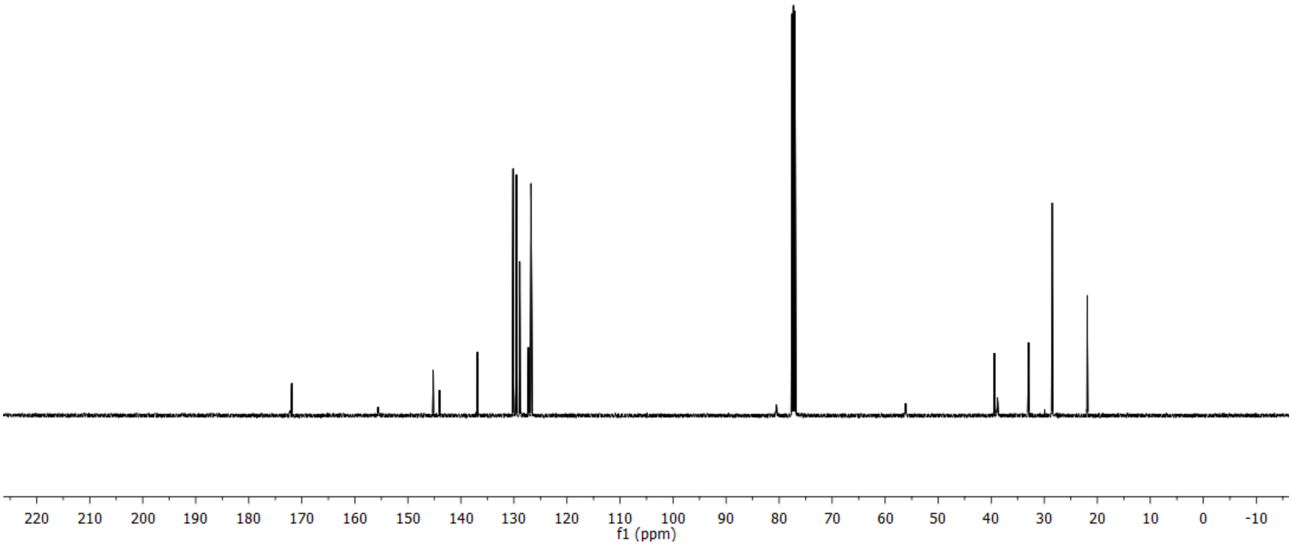

Figure S5. *Cont.*

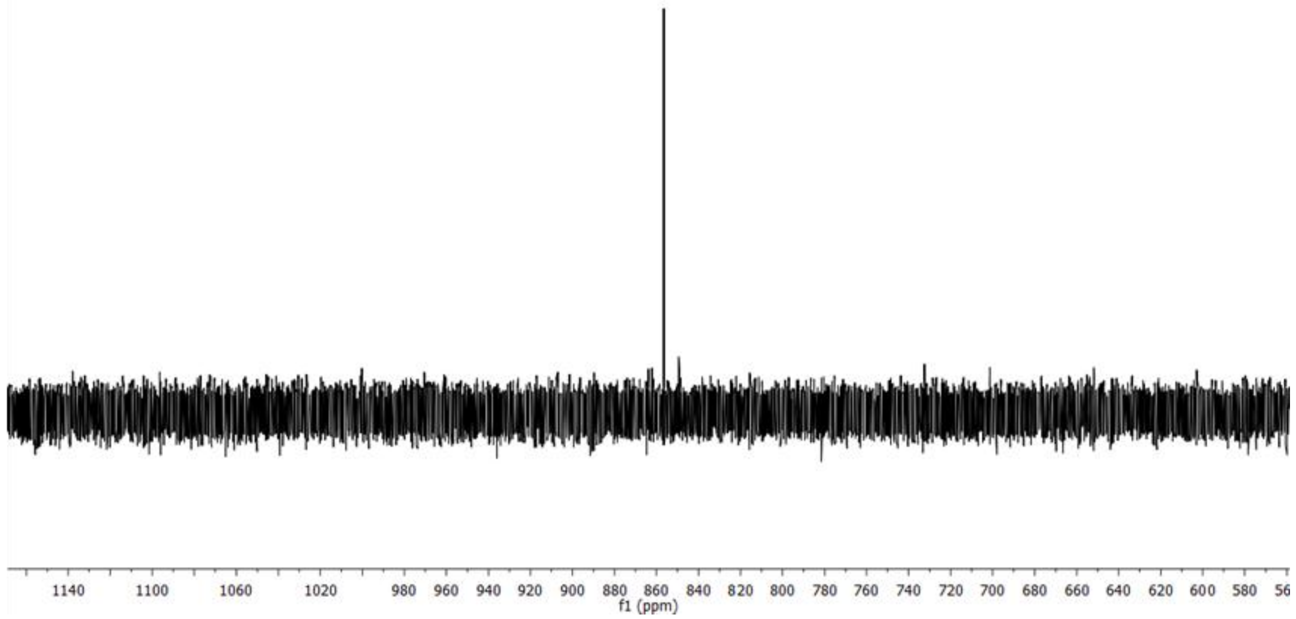

Figure S6. NMR Spectra for Compound **14**.

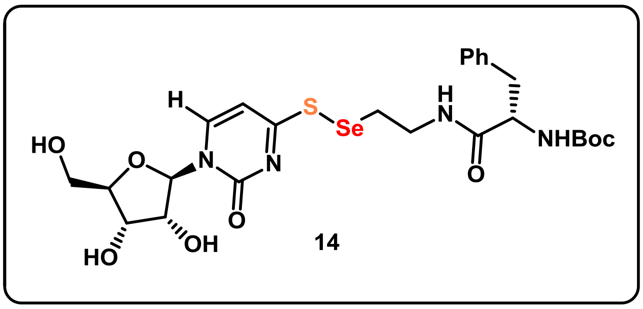

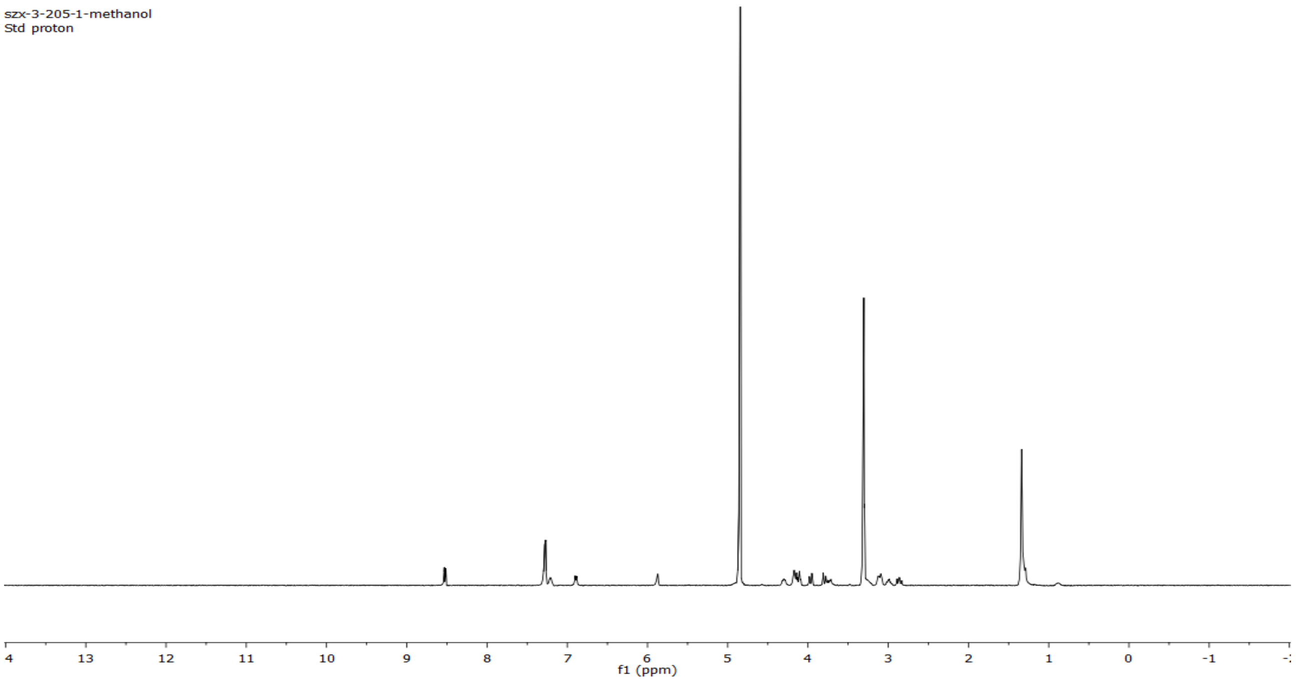

Figure S6. *Cont.*

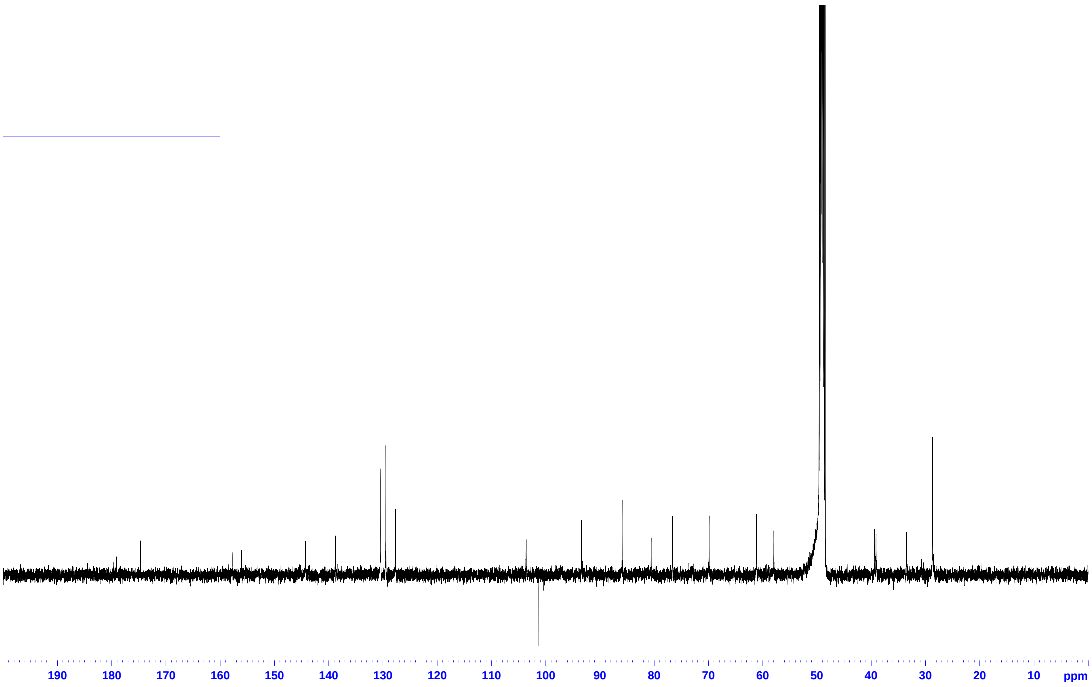

Figure S7. NMR Spectra for Compound **16**.

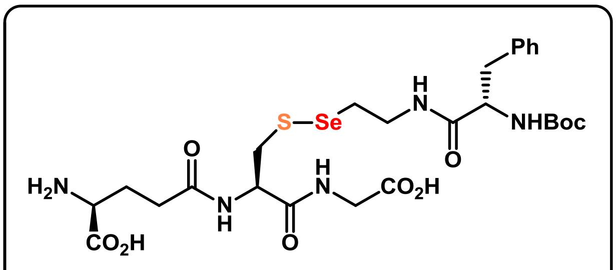

16

Chromatogram of Compound **16**.

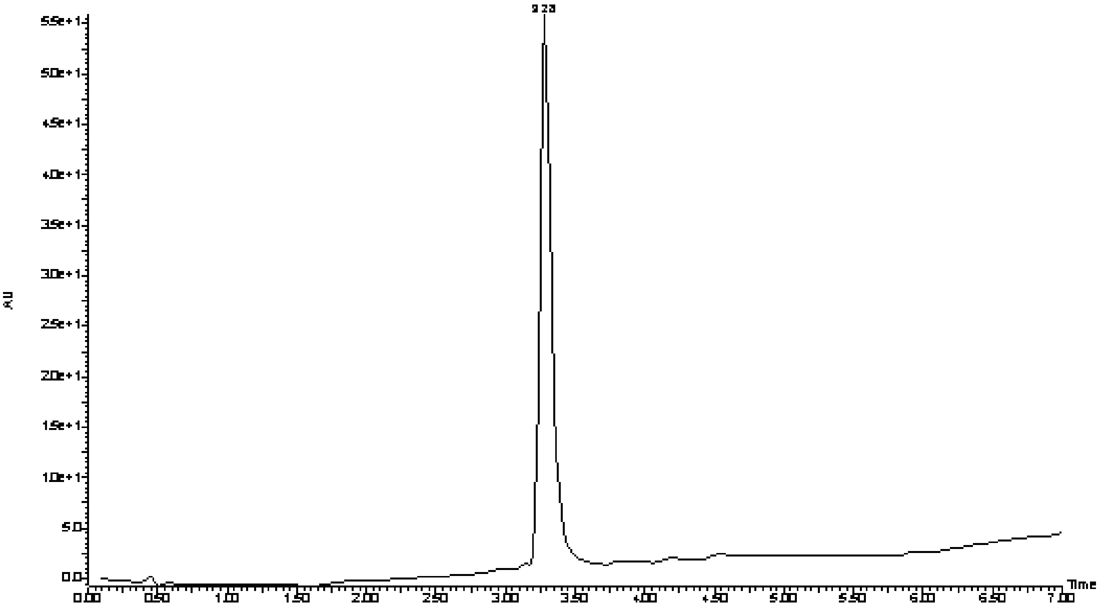

Figure S7. *Cont.*

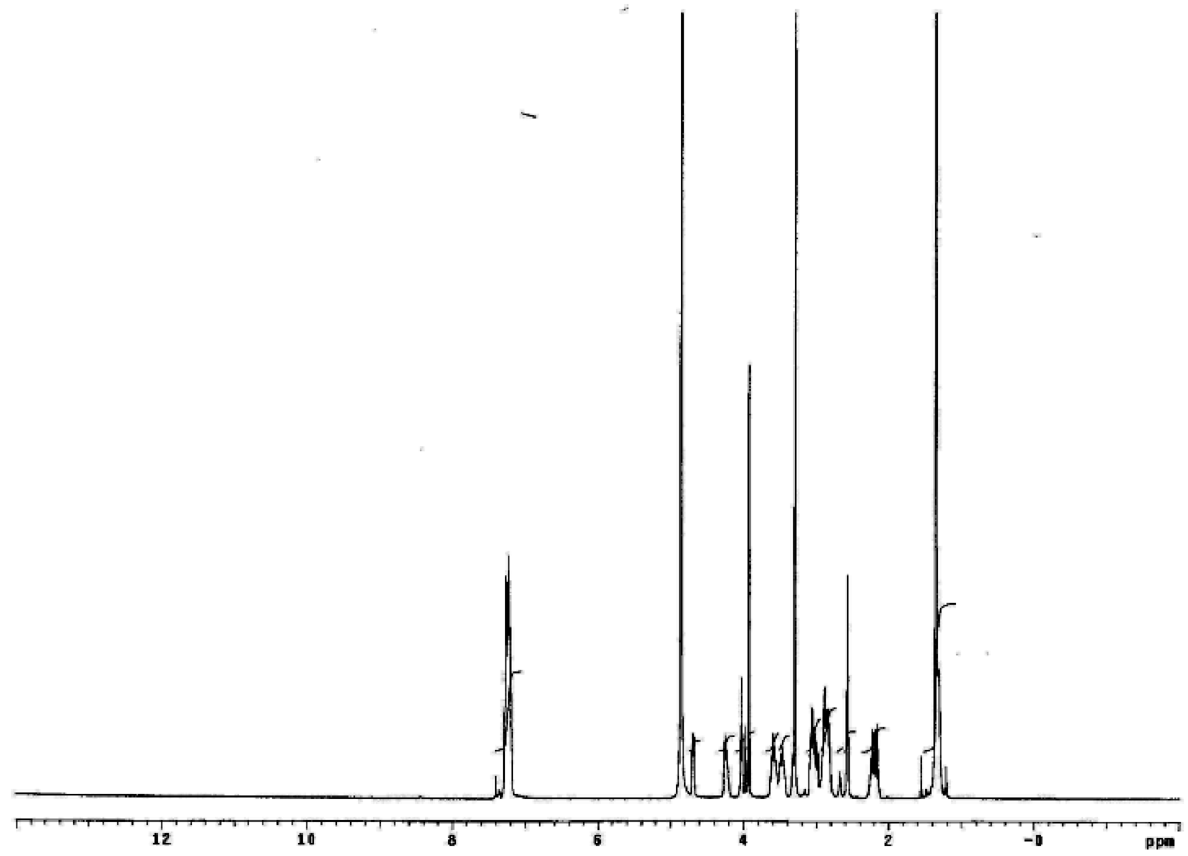

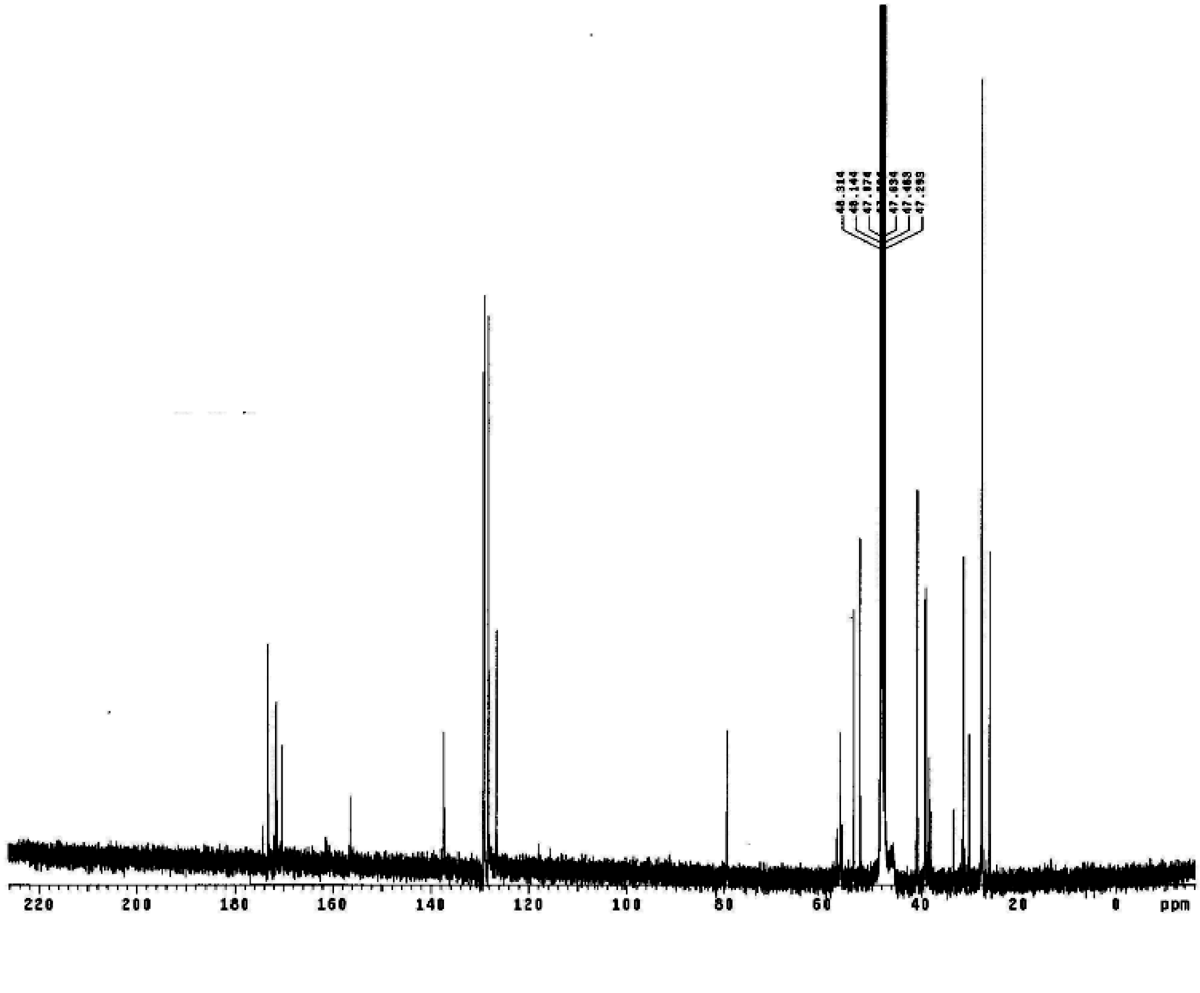

Supplement: Supplementary file 1 [file molecules-18-01963-s001.pdf]
